# Status of Emergency Signal Functions* in Myanmar Hospitals: A Cross-Sectional Survey

**DOI:** 10.5811/westjem.2019.7.43014

**Published:** 2019-10-24

**Authors:** Dong Hyun Seo, Hoon Kim, Kyung Hwan Kim, Junseok Park, Dong Wun Shin, Joon Min Park, Hyunjong Kim, Woochan Jeon, Jung Eon Kim

**Affiliations:** * Na-eun Hospital, Department of Emergency Medicine, Incheon, Korea; † Inje University Ilsan Paik Hospital, Department of Emergency Medicine, Goyang, South Korea

## Abstract

**Introduction:**

Low- and middle-income countries (LMICs) have a large percentage of global mortality and morbidity rates from non-communicable diseases, including trauma. The establishment and development of emergency care systems is crucial for addressing this problem. Defining gaps in the resources and capacity to provide emergency healthcare in LMICs is essential for proper design and operation of ECS (emergency care services) reinforcement programs. Myanmar has particular challenges with road access for providing timely emergency medical care, and a shortage of trained health workers. To examine the ECS capacity in Myanmar, we used the Emergency Care Assessment Tool (ECAT), which features newly developed tools for assessing sentinel conditions and signal functions (key interventions to address morbidity and mortality) in emergency care facilities.

**Methods:**

ECAT is composed of six emergent sentinel conditions and corresponding signal functions. We surveyed a total of nine hospitals in five states in Myanmar. A constructed survey sheet was delivered by e-mail, and follow-up interviews were conducted via messenger to clarify ambiguous answers.

**Results:**

We categorized the nine participating institutions according to predefined criteria: four basic-level hospitals; four intermediate-level; and one advanced-level hospital. All basic hospitals were weak in trauma care, and two of 12 signal functions were unavailable. Half of the intermediate hospitals showed weakness in trauma care, as well as critical care such as shock management. Only half had a separate triage area for patients. In contrast, all signal functions and resources listed in ECAT were available in the advanced-level hospital.

**Conclusion:**

Basic-level facilities in Myanmar were shown to be suboptimal in trauma management, with critical care also inadequate in intermediate facilities. To reinforce signal functions in Myanmar health facilities, stakeholders should consider expanding critical functions in selected lower-level health facilities. A larger scale survey would provide more comprehensive data to improve emergency care in Myanmar.

## INTRODUCTION

Myanmar, formerly Burma, and now administratively designated the Republic of the Union of Myanmar, is a sovereign state in Southeast Asia. Myanmar has a diverse—135 different ethnic groups—population of 53 million according to the United Nations Population Division.^1^ Recently, the military regime that long hampered the country’s development was replaced by a civilian government.^2^ Socioeconomic development in Myanmar lags far behind nearby countries, as does its healthcare system. There are shortcomings in maternal care, pediatric healthcare, and infectious disease treatment, as well as medical accessibility and quality.^3^

Strengthening medical systems by improving the standard of emergency care has been known to reduce the mortality and morbidity from both communicable and non-communicable diseases.^4^,^5^ A large proportion of the global mortality and morbidity rate from various diseases is found in low- and middle-income countries (LMICs). Unfortunately, the emergency care systems required to address these shortcomings are not well established in most LMICs, including Myanmar.^6^ Formal emergency care in Myanmar is only available in hospitals located in urban areas. Rural hospitals can provide only limited emergency care to patients.^7^

While preparing for an international sporting event, the Myanmar government started to formalize efforts to develop a formal emergency medicine (EM) training program.^8^ Apart from the formal EM training program in the capital city, Nay Pyi Taw, frontline healthcare facilities across the country are not capable of providing life-saving emergency care. In most rural hospitals, the outpatient department usually covers emergencies; there is no separate area or facility for emergency treatment. Rural hospitals offer access to few medical specialties with minimal, if any, laboratory services. Public prehospital ambulance transportation service is virtually unavailable in rural areas.^9^

Several tools have been used to evaluate emergency care capability. Most focused primarily on the availability of hardware or infrastructure rather than functional aspects of emergency care.^10^ Some researchers have tried to measure performance of EM practice in resource-limited settings, which has resulted in a demand for a comprehensive EM assessment tool for LMICs.^11^,^12^,^13^ Recently, a novel approach based on work in the field of obstetrics, called sentinel condition and signal function, was adapted for EM by the African Federation for Emergency Medicine (AFEM).^14^, ^15^

Based on this concept, the AFEM developed a standard preliminary tool called the Emergency Care Assessment Tool (ECAT), which has been suggested to be more useful than previous evaluation tools in assessing EM systems.^10^ Our study incorporated the concept of ECAT as a tool to analyze Myanmar’s emergency care systems. We investigated the capability to deliver emergency care in different levels of hospitals located in several regions of Myanmar.

**Population Health Research Capsule**

What do we already know about this issue?

*The higher mortality and morbidity rates in hospitals in low-and middle-income countries often arise from suboptimal emergency care systems*.

What was the research question?

Would implementation of a recently validated Emergency Care Assessment Tool effectively assess emergency care capacity in various levels of Myanmar hospitals?

What was the major finding of the study?

*Basic-level facilities in Myanmar were suboptimal in trauma care, with critical care also inadequate in intermediate-levels hospitals*.

How does this improve population health?

*To reinforce signal functions in Myanmar health facilities, stakeholders should consider expanding critical functions in selected lower-level health facilities*.

## METHODS

**Figure 1:**
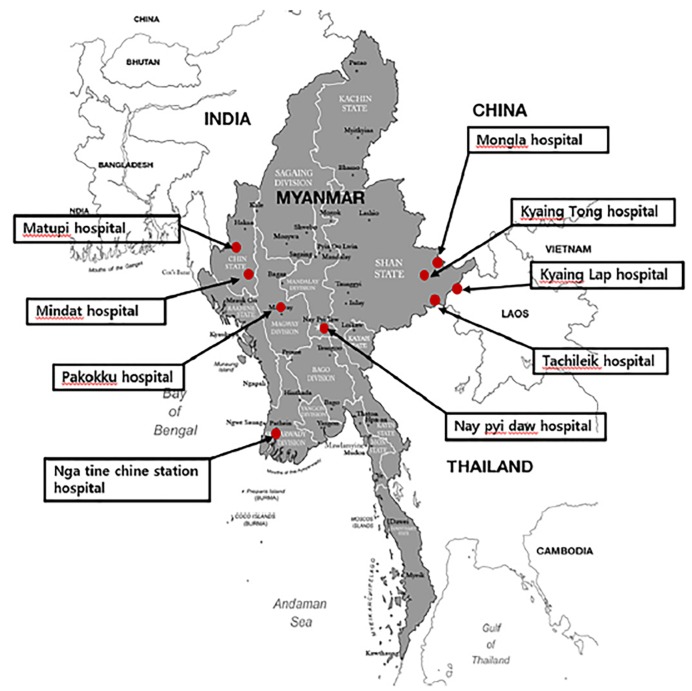
Geographical distribution of hospitals investigated.

This facility-based survey was conducted between February 7, 2018 –April 3, 2018. With the help of two Myanmar doctors and three nurses who were invited to Korea for training, survey sheets were distributed to the doctors in charge of emergency medical care at nine hospitals. Our primary criterion for selecting hospitals was access to e-mail and online messaging, at the time of survey, to allow for our interactions with them. The nine hospitals, including five at which our initial contacts were employed, were scattered in five states in Myanmar, and believed to partially represent both urban and rural regions (Figure 1). The nine hospitals were grouped into three levels, according to the bed capacity of the hospital (fewer than 100 beds, 100–1000 beds, over 1000 beds) and the number of physicians (fewer than five, 5–100, over 100).

Survey sheets were prepared in English using ECAT and delivered to responsible officers by e-mail. ECAT encompasses six sentinel conditions that threaten life (respiratory failure, shock, altered mental status, dangerous fever, severe pain, and trauma), and the related signal functions (key interventions) that alleviate them. The researchers explained the meaning of each question in the survey to the original five Myanmar contacts, and they, in turn, conveyed this information to the Myanmar doctors who took part in the study. In the case of any questions that were initially omitted on the completed surveys, clarification was provided, and the questions were then revisited and answered by the respondents.

The survey included questions about the general status of each hospital, such as the number of staff members, the number of hospital beds, and the annual patient load. The remaining questions addressed the performance of emergency signal functions, the products for signal functions (eg, airway management and defibrillation for resuscitation), and the availability of emergency facility infrastructures. We coded data using standard descriptive analyses with Microsoft Excel 2015 (Seattle, Washington, USA). Qualitative research methods involved thematic analysis of answers.

## RESULTS

All nine hospitals completed the survey sheet. Based on the predefined criteria, four hospitals were classified as basic level, four as intermediate, and one as advanced level, as shown in Table 1. We summarize results by the availability of performances/products/infrastructure in the Supplemental Tables 1–3. Table 2 shows the overall adherence rate to the signal functions.

### Basic-Level Hospitals

In performing signal functions for each of the sentinel conditions, basic-level hospitals were revealed to be weak in trauma care. Among the 12 signal functions related to trauma care that are deemed essential in basic-level hospitals, more than two functions were unavailable at all four hospitals. One hospital could not provide half of the trauma-related essential signal functions [Matupi Hospital–trauma protocol implementation (adult and paediatric), pelvic wrapping, cervical spine immobilization, basic fracture immobilisation (sling, splint, inline immobilisation for other spinal fracture), immediate cooling care for burns, fracture reduction]. None of the four basic-level hospitals had the resources to treat burn patients or provide pelvic wrapping. The survey questions regarding infrastructure revealed that none had a specialized resuscitation area for critical patients, and three of the hospitals did not have a triage area. There was neither trauma protocol nor a cervical immobilization device at any of the hospitals. Most signal functions for the other five sentinel conditions were generally available in these basic-level hospitals, with the exception of treatment for common toxidromes, which only half could provide.

### Intermediate-Level Hospitals

Two of the four intermediate-level hospitals indicated that they could provide all emergency signal functions. The other two hospitals, however, were found to provide a limited set of signal functions. They did not have a trauma protocol nor could they provide reduction for patients with bone fractures. Cervical immobilization, pelvic wrapping, burn care, and treatment of compartment syndrome were also unavailable. Moreover, one hospital could not perform defibrillation or mechanical ventilation support, nor administer intramuscular adrenaline, which is important for cardiopulmonary resuscitation. Two hospitals could insert central venous catheters and gain intraosseous access, which is important in shock management. In terms of resources, only two of the four had a separate triage area for emergency patients. All four hospitals had an isolation room, an obstetric/gynecologic area, and a decontamination room.

### Advanced-Level Hospital

Nay Pyi Taw General Hospital was the only advanced-level hospital in the study. It was able to provide all emergency signal functions, and was equipped with all necessary hardware, with the exception of a fluid warmer for shock treatment.

### Explanation for Non-compliance with Signal Functions

We surveyed hospitals on their reasons for non-compliance with signal functions, asking them to choose from among five possible causal factors. The first was training issues, taking the form of a lack of education. The second factor was related to the lack of availability of appropriate supplies, equipment, and/or drugs. The third pertained to management issues, such as the staff being unfamiliar with the functions, and cases where other equivalent procedures could have handled the conditions. The fourth factor was policy issues, referring to cases where the government or the facility itself does not allow for compliance with the signal functions. The fifth factor was designated as “no indication,” meaning that there was no patient group who needed this function.

Supplemental Table 4 describes the reasons respondents provided on the survey for each unavailable signal function. Inappropriate supplies/equipment/drugs was the most common reason, as might be expected, and shortage of human resources was another causal factor. One intermediate hospital did not agree with the use of emergency signal functions for sentinel conditions, and answered “no indication” as their reason for non-compliance.

## DISCUSSION

It is widely recognized that there is a huge burden caused by trauma and non-communicable diseases in LMICs, where capability for emergency care is believed to be suboptimal.^16^ Many studies have tried to assess the state of emergency care in the health facilities of LMICs. Due to the accessibility issue, most studies examined teaching hospitals located in urban areas. Assessment tools were not standardized and were usually developed by the researchers themselves. Domains for assessment were usually related to the availability of resources, and functional aspects were surveyed with qualitative measures, if any. To our knowledge, this study is the first to survey urban and rural Myanmar hospitals using ECAT, the newly developed objective tool for assessing emergency care in health facilities.

Our study demonstrated that the performance of emergency signal functions in Myanmar hospitals is inadequate, especially in trauma care. Trauma care in LMICs has been regarded as a role for large hospitals, and direct referral to upper-level facilities is a common practice. Burke et al. found that lack of readily accessible equipment for trauma care and shortage of skilled staff were the main reasons for poor quality trauma care in lower-level health facilities in LMICs.^17^ Another study pointed out the limited training opportunities for trauma management in LMICs.^18^ We found similar obstacles to trauma care in Myanmar hospitals, including the unavailability of items necessary for signal functions.

Unlike other LMICs, Myanmar faces a singular geographic and demographic situation. Road conditions are poor. Almost 20 million people live in areas not connected by basic roads. The roads that do exist are unpaved and narrow, contributing to the overall lack of accessibility. The cause of this problem might be found in continuous armed conflicts. Since the independence of Myanmar in 1948, a continuing civil war has devastated the population and infrastructure of the rural areas, which has led to the deterioration of the health status of the country.

**Figure 2:**
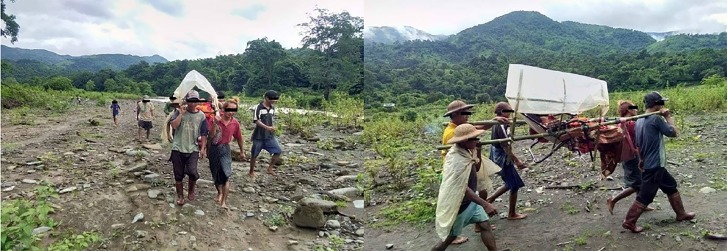
Common transportation method in rural area of Myanmar (photo taken near Matupi Hospital).

In areas dominated by violence, residential zones are located away from road access, and the level of medical care is behind the times. Financial support is also lacking.^19^ For example, a referral and transport from Matupi Hospital to an adjacent upper-level facility takes as long as 16 hours during rainy seasons due to road damage (Figure 2). In this situation, timely management of patients in a critical condition is virtually impossible, and demands for higher levels of emergency care in basic-level facilities can be raised. Moreover, the results of our study show that some intermediate-level hospitals could not provide resuscitation for critical patients due to the lack of advanced airway management, mechanical ventilators, and defibrillation.

Imbalances in the quality of emergency care in both basic- and intermediate-level facilities should be addressed carefully. However, in Myanmar’s special situation where highway infrastructure is lacking and there are problems with long transport times, the ability to administer emergency medical care at a large hospital should be established based on skilled labor and resources. Ouma et al. emphasized that all countries should reach the international benchmark of more than 80% of their populations living within a two-hour travel time to the nearest hospital.^20^ Although it cannot be realized in the near future, measures to alleviate accessibility problems can be applied. Extension of critical signal functions for time-dependent conditions should be considered in selected basic-level facilities. Thorough gap analyses to address existing challenges in remote regions will be helpful for planning. In this regard, ECAT should be validated to include a time factor, such as the referral time to the nearest upper-level facility.

We identified the following urgent issues in need of remediation: 1) improvement of trauma-related signal functions in basic-level facilities; 2) improvement of trauma- and critical care-related signal functions in intermediate level facilities; and 3) implementation of a comprehensive nationwide survey to uncover emergency care deficiencies in rural areas, with emphasis on the time required for referral to higher-level facilities. Our suggestions to address the issues identified in our study can be summarized as relating to the reinforcement of infrastructure and human resources within each level of facility. In addition, prehospital care and care during inter-facility transportation should receive special attention considering the unique context of Myanmar, with its dispersed residences and extremely long transport times.

There has been an effort to establish formal EM in Myanmar. In 2014, the Emergency Medicine Postgraduate Diploma course provided by Australia graduated 18 Myanmar medical officers.^8^ These emergency providers will be an imperative asset to setting up a modern emergency medical care delivery system in Myanmar, although most of them will practice in advanced-level facilities. Measures to build the capacity to respond to medical emergencies in rural areas should be pursued in Myanmar. There have already been efforts to improve first-aid skills among local healthcare workers who have a high degree of understanding of the local context, and to employ them as community emergency responders.^21^

These local healthcare workers are well informed about the population, hygiene, disease distribution, and the geographical and cultural characteristics of the area; thus, they are able to provide essential first aid and find appropriate health facilities for referrals. This practice has been expanded to the concept of out-of-hospital emergency care (OHEC). It refers to a wide range of emergency treatments, from the process of recognizing an emergent care situation, to the initial emergency treatments outside the hospital, and transport to the hospital.^22^ The establishment of OHEC has played a role particularly in LMICs by reducing mortality rates by 80%, especially in trauma cases.^23^

Since 2000, several organizations have implemented the trauma training course (TTC) program with non-physician clinicians (called health workers) in Eastern Myanmar.^24^ The program comprises various skills for carrying out the initial treatment of trauma, taught through simple simulations and feedback. The findings indicated that survival rates improved significantly among major trauma patients following the implementation of this program. We recognize that some skills covered in the TTC, such as surgical airway management, would be relatively dangerous for health workers to perform in the field, and believe that development and implementation of a training program focused on the operation of emergency signal functions would be more practical for the rural context. Those who are trained in this program could act as prehospital emergency care providers, and also aid basic-level facilities to fill the functional gaps identified in this study.

In addition to the above suggestions, a national or provincial strategic plan for reinforcing emergency care in rural areas of Myanmar should be established and implemented. Following a thorough investigational survey, essential resources for each level of health facility should be supplemented. Public education to recognize emergency conditions is another area to be strengthened. In many LMICs, including Myanmar, folk remedies are still commonly attempted before people seek medical attention, especially in the field of obstetrics and gynecology.^25^ Recognizing the need for emergency care is crucial because it is the first step leading the patient to the emergency medical care system. Community education should play an important role in preventing delays in the detection of emergency situations.^26^ Traditional medicine providers have been the first to participate in this training thus far, and it has been reported to be effective.^27^

## LIMITATIONS

One limitation of the present study is the possibility of recall bias because we collected the data retrospectively. To minimize this bias, we selected five hospitals first, each of which had a key staff member whom we could contact frequently in a direct way. The other four hospitals were contacted via e-mail as a result of guidance we received from our initial five participants, who put us in direct contact with these additional research hospitals. Another limitation of our study is selection bias, given that the research hospitals taking part were not randomly selected. While the research hospitals were dispersed across various rural areas of Myanmar, they cannot be taken to represent each region,; however, they do provide a snapshot of the different levels of health facilities in Myanmar, and provide us with the basis for planning a more comprehensive survey on a larger scale in the future.

## CONCLUSION

Our study revealed that emergency signal functions in basic-level facilities in rural areas of Myanmar are suboptimal, specifically in trauma care. Additionally, critical care in intermediate-level facilities is also compromised, and should receive more attention. A survey at the provincial or national level is needed to address existing gaps in the functionality of emergency signal functions. Stakeholders related to the emergency medical care project should adopt the results of this survey and plan their project in such a way as to improve emergency signal functions within each level of facility. In particular, it is necessary to consider strengthening selected basic-level facilities in remote areas, to overcome unacceptably long transport times.

## Supplementary Information



## Figures and Tables

**Table 1:** Overview of hospitals included in a survey of emergency medical care delivery in Myanmar.

Name	Bed	Number of Doctors	Number of nurses	Location	Annual OPD visit number	Annual admissions
Basic						
Kyaing Lap Station Hospital	16	1	5	Eastern Shan State	3353	627
Matupi General Hospital	50	2	26	Chin State	10847	1997
Mong La General Hospital	50	2	21	Eastern Shan State	17375	1661
Nga Tine Chine Station Hospital	16	1	5	Ayeyarwaddy region	4609	2382
Intermediate						
MinDat General Hospital	100	9	70	Chin State	16770	3100
Kyaing Tong General Hospital	200	30	199	Eastern Shan State	45954	11082
Tachileik General Hospital	100	23	78	Eastern Shan State	25826	8513
Pakokku General Hospital	200	72	182	MaGway region	34789	26919
Advanced						
Nay Pyi Daw General Hospital	1000	238	402	Nay Pyi Daw region	208573	28819

*OPD*, outpatient department.

**Table 6:** Adherence rate to signal function for each sentinel condition in each category of hospitals.

	Basic	Intermediate	Advanced
Performance analysis			
Respiratory failure	92%	91%	100%
Shock	92%	92%	100%
Altered mental status	100%	93%	100%
Severe pain	92%	98%	100%
Trauma	71%	83%	100%
Dangerous fever	81%	100%	100%
Product analysis			
General products	98%	100%	100%
Respiratory failure products	94%	91%	100%
Shock products	100%	75%	93%
Altered mental status	100%	100%	100%
Severe pain/trauma and burns	75%	100%	100%
Dangerous fever	none	100%	100%
Infrastructure analysis			
Adherence rate of total infrastructure	69%	86%	100%
