# Supplementary material for: Status of Emergency Signal Functions in Myanmar Hospitals: A Cross-Sectional Survey
Source: West J Emerg Med. 2019 Oct 24;20(6):903–9. doi: 10.5811/westjem.2019.7.43014 (PMC6860398; doi:10.5811/westjem.2019.7.43014)
Supplement: Supplementary file 1 [file wjem-20-903-s001.docx]

**Supplemental Table 1**. Performance of signal functions for each sentinel condition.

| Performance Investigation | | BASIC | | | | INTERMEDIATE | | | | ADVANCED |
| --- | --- | --- | --- | --- | --- | --- | --- | --- | --- | --- |
| Sentinel conditions | Items | kyaing lap | Matupi | Mong La | Mga | Mindat | Kyaing tong | Tachileik | PakhotCu | Nay Pyi Taw |
| Respiratory failure | Manual manoeuvres | Y | Y | Y | Y | Y | Y | Y | Y | Y |
|  | Relief of obstruction | Y | Y | Y | Y | Y | Y | Y | Y | Y |
|  | Rescue breathing | Y | Y | Y | Y | Y | Y | Y | Y | Y |
|  | Three-way dressing | Y | Y | Y | **N** | Y | Y | Y | **N** | Y |
|  | Insertion of oral airway | **N** | Y | **N** | Y | Y | Y | Y | Y | Y |
|  | Bag-valve mask ventilation | Y | Y | Y | Y | Y | Y | Y | Y | Y |
|  | Needle decompression |  |  |  |  | Y | Y | Y | **N** | Y |
|  | Non-definitive advanced airway with supraglottic device |  |  |  |  | Y | Y | Y | **N** | Y |
|  | Administration of critical therapeutics |  |  |  |  | Y | Y | Y | Y | Y |
|  | Oxygen administration |  |  |  |  | Y | Y | Y | Y | Y |
|  | Use of suction |  |  |  |  | Y | Y | Y | Y | Y |
|  | Definitive advanced airway |  |  |  |  | Y | Y | Y | Y | Y |
|  | Radiograph interpretation |  |  |  |  | Y | Y | Y | Y | Y |
|  | Mechanical ventilation: invasive and non-invasive ventilation |  |  |  |  | **N** | Y | Y | **N** | Y |
|  | Chest tube insertion |  |  |  |  |  |  |  |  | Y |
|  | Surgical airway |  |  |  |  |  |  |  |  | Y |
| Shock | Physical manoeuvres for control of haemorrhage | Y | Y | Y | Y | Y | Y | Y | Y | Y |
|  | Arterial tourniquet | Y | Y | Y | Y | Y | Y | Y | Y | Y |
|  | Pelvic wrapping | **N** | **N** | **N** | Y | Y | Y | Y | **N** | Y |
|  | Administration of oral rehydration | Y | Y | Y | Y | Y | Y | Y | Y | Y |
|  | Fracture splinting | Y | Y | Y | Y | Y | Y | Y | Y | Y |
|  | Treatment with antimicrobial agents | Y | Y | Y | Y | Y | Y | Y | Y | Y |
|  | Traction splinting |  |  |  |  | Y | Y | Y | Y | Y |
|  | Peripheral percutaneous intravenous access |  |  |  |  | Y | Y | Y | Y | Y |
|  | Intraosseous access |  |  |  |  | **N** | Y | Y | **N** | Y |
|  | Venous cutdown |  |  |  |  | Y | Y | Y | Y | Y |
|  | Intravenous fluid administration |  |  |  |  | Y | Y | Y | Y | Y |
|  | Administration of critical therapeutics |  |  |  |  | Y | Y | Y | Y | Y |
|  | Packing and suturing for haemorrhage control |  |  |  |  | Y | Y | Y | Y | Y |
|  | External defibrillation/cardioversion |  |  |  |  | **N** | Y | Y | Y | Y |
|  | ECG interpretation |  |  |  |  | Y | Y | Y | Y | Y |
|  | Needle decompression |  |  |  |  | Y | Y | Y | **N** | Y |
|  | Administration of intramuscular adrenaline |  |  |  |  | Y | Y | Y | **N** | Y |
|  | Radiograph interpretation |  |  |  |  | Y | Y | Y | Y | Y |
|  | Ultrasonography |  |  |  |  | Y | Y | Y | Y | Y |
|  | Pathogen-screened blood transfusion |  |  |  |  | Y | Y | Y | Y | Y |
|  | Administration of parenteral medication that requires advanced monitoring (eg, vasopressor agents, thrombolytics) |  |  |  |  |  |  |  |  | Y |
|  | Central venous access |  |  |  |  |  |  |  |  | Y |
|  | Chest tube placement |  |  |  |  |  |  |  |  | Y |
|  | Pericardiocentesis |  |  |  |  |  |  |  |  | Y |
| Altered mental status | Protection from secondary injury | Y | Y | Y | Y | Y | Y | Y | Y | Y |
|  | Glucose monitoring and/or administration of glucose | Y | Y | Y | Y | Y | Y | Y | Y | Y |
|  | Administration of benzodiazepines for seizure or sedation | Y | Y | Y | Y | Y | Y | Y | Y | Y |
|  | Rule out organic causes of altered mental status | Y | Y | Y | Y | Y | Y | Y | **N** | Y |
|  | Administration of appropriate therapeutics for acute psychiatric illness | Y | Y | Y | Y | Y | Y | Y | **N** | Y |
|  | Administration of empiric antimicrobial agents if febrile | Y | Y | Y | Y | Y | Y | Y | **N** | Y |
|  | Magnesium sulfate for pregnant patients with seizure |  |  |  |  | Y | Y | Y | Y | Y |
|  | Laboratory investigations |  |  |  |  | Y | Y | Y | Y | Y |
|  | Administration of insulin for hyperglycaemia |  |  |  |  | Y | Y | Y | Y | Y |
|  | Administration of locally appropriate antidote/antivenom as clinically appropriate for toxic causes |  |  |  |  |  |  |  |  | Y |
|  | Lumbar puncture |  |  |  |  | Y | Y | Y | Y | Y |
|  | Administration of therapeutics for appropriate BP management |  |  |  |  | Y | Y | Y | Y | Y |
|  | ECG interpretation for metabolic abnormalities |  |  |  |  | Y | Y | Y | Y | Y |
|  | CT interpretation |  |  |  |  |  |  |  |  | Y |
| Severe pain | Administration of an analgesic agent | Y | Y | Y | Y | Y | Y | Y | Y | Y |
|  | Administration of aspirin for chest pain | Y | Y | Y | **N** | Y | Y | Y | Y | Y |
|  | Urine dipstick | Y | **N** | Y | Y | Y | Y | Y | **N** | Y |
|  | Administration of oral rehydration | Y | Y | Y | **N** | Y | Y | Y | Y | Y |
|  | Paracentesis | Y | Y | Y | N | Y | Y | Y | Y | Y |
|  | Placement of Foley catheter for urinary outlet obstruction | Y | Y | Y | Y | Y | Y | Y | Y | Y |
|  | Intravenous fluid administration |  |  |  |  | Y | Y | Y | Y | Y |
|  | ECG interpretation |  |  |  |  | Y | Y | Y | Y | Y |
|  | Radiograph interpretation |  |  |  |  | Y | Y | Y | Y | Y |
|  | Ultrasound interpretation |  |  |  |  | Y | Y | Y | Y | Y |
|  | Definitive surgical services |  |  |  |  | Y | Y | Y | Y | Y |
|  | CT interpretation |  |  |  |  |  |  |  |  | Y |
| Trauma | Trauma protocol implementation (adult and paediatric) | **N** | **N** | **N** | Y | **N** | Y | Y | Y | Y |
|  | Physical manoeuvres for control of haemorrhage | Y | Y | Y | Y | Y | Y | Y | Y | Y |
|  | Arterial tourniquet | Y | Y | Y | Y | Y | Y | Y | Y | Y |
|  | Pelvic wrapping | **N** | **N** | **N** | **N** | **N** | Y | Y | **N** | Y |
|  | Appropriate wound care | Y | Y | Y | Y | Y | Y | Y | Y | Y |
|  | Protect from secondary injury | Y | Y | Y | Y | Y | Y | Y | Y | Y |
|  | Cervical spine immobilisation | Y | **N** | Y | **N** | **N** | Y | Y | Y | Y |
|  | Basic fracture immobilisation (sling, splint, inline immobilisation for other spinal fracture) | Y | **N** | Y | Y | **N** | Y | Y | Y | Y |
|  | Immediate cooling care for burns | **N** | **N** | N | **N** | **N** | Y | Y | **N** | Y |
|  | Fracture reduction | Y | **N** | Y | Y | **N** | Y | Y | Y | Y |
|  | Antibiotic administration for open fracture | Y | Y | Y | Y | Y | Y | Y | Y | Y |
|  | Tetanus vaccination | Y | Y | Y | Y | Y | Y | Y | Y | Y |
|  | Traction splinting |  |  |  |  | Y | Y | Y | Y | Y |
|  | Radiograph interpretation |  |  |  |  | Y | Y | Y | Y | Y |
|  | Fasciotomy for compartment syndrome |  |  |  |  | **N** | Y | Y | **N** | Y |
|  | Packing and suturing for haemorrhage control |  |  |  |  | Y | Y | Y | Y | Y |
|  | Peripheral percutaneous intravenous access |  |  |  |  | Y | Y | Y | Y | Y |
|  | Intraosseous access |  |  |  |  | **N** | Y | Y | **N** | Y |
|  | Venous cutdown |  |  |  |  | **Y** | Y | Y | Y | Y |
|  | Intravenous fluid administration |  |  |  |  | Y | Y | Y | Y | Y |
|  | Escharotomy |  |  |  |  | **N** | Y | Y | Y | Y |
|  | Needle decompression |  |  |  |  | Y | Y | Y | **N** | Y |
|  | Definitive surgical services |  |  |  |  | **N** | Y | Y | Y | Y |
|  | Rabies IVIG/vaccination |  |  |  |  |  |  |  |  | Y |
|  | Chest tube placement |  |  |  |  |  |  |  |  | Y |
|  | Auto-transfusion from chest tubes |  |  |  |  |  |  |  |  | Y |
|  | Thoracotomy |  |  |  |  |  |  |  |  | Y |
|  | CT interpretation |  |  |  |  |  |  |  |  | Y |
| Dangerous fever | Management of extremes of temperature | Y | Y | Y | Y | Y | Y | Y | Y | Y |
|  | Treatment with antimicrobial agent | Y | Y | Y | Y | Y | Y | Y | Y | Y |
|  | Sepsis protocol implementation (paediatric and adult) | Y | **N** | Y | Y | Y | Y | Y | Y | Y |
|  | Therapeutics for sympathomimetic toxidromes or ethanol withdrawal | Y | **N** | Y | **N** | Y | Y | Y | N | Y |
|  | Intravenous fluid administration |  |  |  |  | Y | Y | Y | Y | Y |
|  | Source control with bedside techniques (eg, abscess, empyema) |  |  |  |  | Y | Y | Y | Y | Y |
|  | Source control requiring operating theatre (deep abscess) |  |  |  |  | Y | Y | Y | Y | Y |
|  | Administration of parenteral medication that requires advanced monitoring (eg, vasopressor agents) |  |  |  |  |  |  |  |  | Y |
|  | Lumbar puncture |  |  |  |  |  |  |  |  | Y |
| *Gray zone: essential - the designated function should be assured at the stated level of the health facility in all cases.  *Non-gray zone: desirable - the designated function represents an increased capability that augments the probability of a successful outcome of appropriate emergency care.  *Blank zone - items that are not needed in the step.  *BP*, blood pressure; *ECG*, electrocardiogram; *CT*, computed tomography. | | | | | | | | | | |

**Supplemental Table 2.** Availability of equipment for performing signal functions.

| Product investigation | | BASIC | | | | INTERMEDIATE | | | | ADVANCED |
| --- | --- | --- | --- | --- | --- | --- | --- | --- | --- | --- |
| Signal function | Facility product requirements | kyaing lap | Matupi | Mong La | Nga | Mindat | Kyaing tong | Tachileik | PakhotCu | Nay Pyi Taw |
| General products | Stethoscope | Y | Y | Y | Y | Y | Y | Y | Y | Y |
|  | Clock | Y | Y | Y | Y | Y | Y | Y | Y | Y |
|  | Non-invasive BP monitoring devise including paediatric and adult cuffs | Y | Y | Y | Y | Y | Y | Y | Y | Y |
|  | Thermometer, including low reading capability | Y | Y | Y | Y | Y | Y | Y | Y | Y |
|  | Glucometer | Y | Y | Y | Y | Y | Y | Y | Y | Y |
|  | Paediatric scale | Y | Y | Y | Y | Y | Y | Y | Y | Y |
|  | Gloves | Y | Y | Y | Y | Y | Y | Y | Y | Y |
|  | Personal protective equipment (eg, surgical masks, gowns) | Y | Y | Y | Y | Y | Y | Y | Y | Y |
|  | Scalpels | Y | Y | Y | Y | Y | Y | Y | Y | Y |
|  | Crutches | Y | **N** | Y | Y | Y | Y | Y | Y | Y |
|  | Documentation | Y | Y | Y | Y | Y | Y | Y | Y | Y |
|  | N95 mask |  |  |  |  | Y | Y | Y | Y | Y |
|  | Lab collection tubes |  |  |  |  | Y | Y | Y | Y | Y |
|  | Pulse oximeter with adult and paediatric probes |  |  |  |  | Y | Y | Y | Y | Y |
|  | Plaster of Paris |  |  |  |  | Y | Y | Y | Y | Y |
|  | Xeroform |  |  |  |  | Y | Y | Y | Y | Y |
|  | Sterile surgical protective equipment |  |  |  |  | Y | Y | Y | Y | Y |
| Respiratory failure products | Water-soluble lubricant | Y | Y | Y | Y | Y | Y | Y | Y | Y |
|  | 10 mL syringe | Y | Y | Y | Y | Y | Y | Y | Y | Y |
|  | Tape | Y | Y | Y | Y | Y | Y | Y | Y | Y |
|  | Meconium adaptor/aspirator | Y | Y | Y | Y | Y | Y | Y | **N** | Y |
|  | Gauze | Y | Y | Y | Y | Y | Y | Y | Y | Y |
|  | Nasogastric tube | Y | Y | Y | Y | Y | Y | Y | Y | Y |
|  | Nebulisation equipment | Y | Y | Y | Y | Y | Y | Y | Y | Y |
|  | Oropharyngeal airways, paediatric and adult | N | Y | Y | **N** | Y | Y | Y | Y | Y |
|  | Nasopharyngeal airways, paediatric and adult | Y | Y | Y | **N** | Y | Y | Y | Y | Y |
|  | Bag-valve mask | Y | Y | Y | Y | Y | Y | Y | Y | Y |
|  | Oxygen concentrator | **N** | Y | Y | Y | Y | Y | Y | Y | Y |
|  | Oxygen | Y | Y | Y | Y | Y | Y | Y | Y | Y |
|  | Oxygen masks |  |  |  |  | Y | Y | Y | Y | Y |
|  | Supraglottic airway |  |  |  |  | Y | Y | Y | Y | Y |
|  | Laryngoscope set with adult and paediatric blades, spare bulb and spare battery |  |  |  |  | Y | Y | Y | Y | Y |
|  | Tracheal tubes, paediatric and adult |  |  |  |  | **N** | Y | Y | Y | Y |
|  | Non-auscultatory endotracheal tube assessment |  |  |  |  | **N** | Y | Y | Y | Y |
|  | Stylets |  |  |  |  | **N** | Y | Y | Y | Y |
|  | Gum elastic bougie, adult and paediatric |  |  |  |  | **N** | Y | Y | **N** | Y |
|  | Suction device and suction catheters, with rigid and flexible tips |  |  |  |  | Y | Y | Y | Y | Y |
|  | 14-gauge catheters (needle decompression) |  |  |  |  | Y | Y | Y | **N** | Y |
|  | Ventilator |  |  |  |  | **N** | Y | Y | Y | Y |
|  | Cricothyroidotomy set |  |  |  |  | Y | Y | Y | Y | Y |
|  | Tracheostomy tubes, paediatric and adult |  |  |  |  | Y | Y | Y | Y | Y |
|  | Chest thoracostomy set |  |  |  |  |  |  |  |  | Y |
| Shock products | Sutures | Y | Y | Y | Y | Y | Y | Y | Y | Y |
|  | Basic surgical kit | Y | Y | Y | Y | Y | Y | Y | Y | Y |
|  | Sharps container | Y | Y | Y | Y | Y | Y | Y | Y | Y |
|  | IV sets and appropriate strapping, including blood administration sets, paediatric and adult |  |  |  |  | Y | Y | Y | Y | Y |
|  | Syringes (1–50 mL) and needles (assorted gauge) |  |  |  |  | Y | Y | Y | Y | Y |
|  | Paediatric and adult intraosseous access |  |  |  |  | **N** | Y | Y | **N** | Y |
|  | Umbilical vein catheters |  |  |  |  | Y | Y | Y | Y | Y |
|  | Drip stand or equivalent hanging device |  |  |  |  | Y | Y | Y | Y | Y |
|  | Monitor/defibrillator or automated external defibrillator with conductive paste or pads, paddles, electrodes |  |  |  |  | **N** | Y | Y | Y | Y |
|  | 12-lead ECG |  |  |  |  | Y | Y | Y | Y | Y |
|  | Cardiac arrest board |  |  |  |  | **N** | **N** | **N** | **N** | Y |
|  | Fluid warmer |  |  |  |  | **N** | Y | Y | **N** | **N** |
|  | Packs and lines for central venous access |  |  |  |  | **N** | Y | Y | **N** | Y |
|  | High-flow infusion catheters, 8.5 |  |  |  |  | **N** | Y | Y | **N** | Y |
| Altered mental status | Blood glucose monitor | Y | Y | Y | Y | Y | Y | Y | Y | Y |
|  | Collection tubes |  |  |  |  | Y | Y | Y | Y | Y |
|  | CT |  |  |  |  |  |  |  |  | Y |
| Severe pain, trauma and burns | Pregnancy testing kits | Y | Y | Y | Y | Y | Y | Y | Y | Y |
|  | C-spine immobilisation, restraining devices | **N** | **N** | **N** | **N** | Y | Y | Y | Y | Y |
|  | Blankets and towel rolls | Y | Y | Y | Y | Y | Y | Y | Y | Y |
|  | Urinary catheters, paediatric and adult | Y | Y | Y | Y | Y | Y | Y | Y | Y |
|  | Limb traction device |  |  |  |  | Y | Y | Y | Y | Y |
|  | Ultrasound |  |  |  |  | Y | Y | Y | Y | Y |
| Dangerous fever | Lumbar puncture kit |  |  |  |  | Y | Y | Y | Y | Y |
| *Gray zone : essential - the designated function should be assured at the stated level of the health facility in all cases.  *Non-gray zone : desirable - the designated function represents an increased capability that augments the probability of a successful outcome of appropriate emergency care.  *Blank zone - items that are not needed in the step  *BP*, blood pressure; *mL*, milliliter; *IV*, intravenous; *ECG*, electrocardiogram; *CT*, computed tomography | | | | | | | | | | |

**Supplemental Table 3.** Status of emergency facility infrastructure in each hospital.

| Infrastructure Investigation | BASIC | | | | INTERMEDIATE | | | | ADVANCED |
| --- | --- | --- | --- | --- | --- | --- | --- | --- | --- |
| Item | kyaing lap | Matupi | Mong La | Nga | Mindat | Kyaing tong | Tachileik | PakhotCu | Nay Pyi Taw |
| Safety considerations | Y | Y | Y | Y | Y | Y | Y | Y | Y |
| Vehicle accessible | **N** | Y | **N** | Y | Y | Y | Y | Y | Y |
| Triage area with multiple-patient capability | **N** | **N** | Y | **N** | **N** | Y | Y | **N** | Y |
| Isolation room | **N** | Y | Y | Y | Y | Y | Y | Y | Y |
| Obstetric/gynaecology area | Y | Y | Y | Y | Y | Y | Y | Y | Y |
| Waiting area for family | Y | Y | Y | Y | Y | Y | Y | **N** | Y |
| Quiet space for family discussions | Y | Y | Y | Y | Y | Y | Y | **N** | Y |
| Safe area for victims of intimate partner violence | Y | **N** | Y | Y | **N** | Y | Y | **N** | Y |
| Dirty utility room | Y | Y | Y | Y | Y | Y | Y | **N** | Y |
| Point-of-care laboratory services | **N** | Y | Y | **N** | Y | Y | Y | **N** | Y |
| Ambulance accessible | **N** | Y | **N** | Y | Y | Y | Y | Y | Y |
| 24-hour services |  |  |  |  | Y | Y | Y | Y | Y |
| Paediatric area |  |  |  |  | Y | Y | Y | Y | Y |
| Procedure room |  |  |  |  | Y | Y | Y | Y | Y |
| Decontamination area |  |  |  |  | Y | Y | Y | Y | Y |
| Education/conference room |  |  |  |  | Y | Y | Y | Y | Y |
| Pharmaceutical dispensing |  |  |  |  | Y | Y | Y | **N** | Y |
| Pharmacist-staffed pharmacy near emergency department |  |  |  |  | Y | Y | Y | **N** | Y |
| Radiograph in immediate proximity |  |  |  |  | Y | Y | Y | Y | Y |
| Ultrasound |  |  |  |  | Y | Y | Y | Y | Y |
| 24-hour laboratory services |  |  |  |  | Y | Y | Y | Y | Y |
| Specialised resuscitation area | **N** | **N** | **N** | **N** | Y | Y | Y | Y | Y |
| Safe psychiatric room |  |  |  |  |  |  |  |  | Y |
| Management centre with communication |  |  |  |  |  |  |  |  | Y |
| Computed tomography |  |  |  |  |  |  |  |  | Y |
| Ophthalmological area |  |  |  |  | **N** | Y | **N** | Y | Y |
| Ear, nose, throat area |  |  |  |  | **N** | Y | **N** | Y | Y |
| Dental area |  |  |  |  | Y | Y | Y | Y | Y |
| *Gray zone : essential - the designated function should be assured at the stated level of the health facility in all cases.  *Non-gray zone : desirable - the designated function represents an increased capability that augments the probability of a successful outcome of appropriate emergency care.  *Blank zone - items that are not needed in the step | | | | | | | | | |

**Supplemental Table 4.** The reason for non-compliance to signal functions.

|  | Items | Did the procedures related to each item performed in your institution over the past year? (N: no/Y: yes) | If not performed ('N' in previous question), why? (Choose the most appropriate reason) 1.Training issues 2.Supplies, equipment, drug issue 3.Management issue 4.Policy issues 5.No indication |
| --- | --- | --- | --- |
| **Kyaing lap** |  |  |  |
| Respiratory failure | Insertion of oral airway | N | 2 |
| Severe pain | Urine dipstick | N | 2 |
| Shock | Pelvic wrapping | N | 2 |
| Trauma | Trauma protocol implementation (adult and paediatric) | N | 2,3 |
|  | Pelvic wrapping | N | 2 |
|  | Cervical spine immobilization | N | 2 |
|  | Immediate cooling care for burns | N | 2,3,4 |
|  |  |  |  |
| **Matupi** |  |  |  |
| Shock | Pelvic wrapping | N | 2 |
| Severe pain | Urine dipstick | N | 2 |
| Trauma | Trauma protocol implementation (adult and paediatric) | N | 3 (no protocol) |
|  | Pelvic wrapping | N | 2 |
|  | Cervical spine immobilisation | N | 2 |
|  | Immediate cooling care for burns | N | 2 |
| Dangerous fever | Sepsis protocol implementation (paediatric and adult) | N | 3 (no protocol) |
|  | Therapeutics for sympathomimetic toxidromes or ethanol withdrawal | N | 3 (no for toxidromes) |
| **Mong La** |  |  |  |
| Severe pain | Urine dipstick | N | 2 |
| Shock | Pelvic wrapping | N | 2 |
| Trauma | Trauma protocol implementation (adult and paediatric) | N | 2,3 |
|  | Pelvic wrapping | N | 2 |
|  | Cervical spine immobilization | N | 2 |
|  | Immediate cooling care for burns | N | 2,3,4 |
| **Nga** |  |  |  |
| Respiratory failure | Three-way dressing | N | 2 |
| Shock | Pelvic wrapping | N | 2 |
| Severe pain | Administration of oral rehydration | N | 3 |
|  | Paracentesis | N | 3 |
| Trauma | Pelvic wrapping | N | 1 |
|  | Cervical spine immobilisation | N | 2 |
|  | Immediate cooling care for burns | N | 2 |
| Dangerous fever | Therapeutics for sympathomimetic toxidromes or ethanol withdrawal | N | 3 |
|  |  |  |  |
| **Mindat** |  |  |  |
| Respiratory failure | Mechanical ventilation: invasive and non-invasive ventilation | N | 2 |
| Shock | Intraosseous access | N | 2 (no support) |
|  | External defibrillation/cardioversion | N | 2 (no support) |
| Trauma | Trauma protocol implementation (adult and paediatric) | N | 3 (no protocol) |
|  | Pelvic wrapping | N | 2 |
|  | Cervical spine immobilisation | N | 2 |
|  | Basic fracture immobilisation (sling, splint, inline immobilisation for other spinal fracture) | N | 2 |
|  | Immediate cooling care for burns | N | 2 |
|  | Fracture reduction | N | 2 |
|  | Fasciotomy for compartment syndrome | N | 3 (no patient before and no surgeon) |
|  | Intraosseous access | N | 2 |
|  | Venous cutdown | N | 2 |
|  | Escharotomy | N | 3 (no patient before and no surgeon) |
|  | Definitive surgical services | N | 3 (no surgeon) |
| **Pakokku** |  |  |  |
| Respiratory failure | Three-way dressing | N | 2 |
|  | Needle decompression | N | 5 |
|  | Non-definitive advanced airway with supraglottic device | N | 2 |
|  | Mechanical ventilation: invasive and non-invasive ventilation | N | 2 |
| Shock | Pelvic wrapping | N | 2 |
|  | Intraosseous access | N | 2 |
|  | Needle decompression | N | 5 |
|  | Administration of intramuscular adrenaline | N | 5 |
| Altered mental status | Rule out organic causes of altered mental status | N | 5 |
|  | Administration of appropriate therapeutics for acute psychiatric illness | N | 5 |
|  | Administration of empiric antimicrobial agents if febrile | N | 5 |
| Severe pain | Urine dipstick | N | 2 |
| Trauma | Pelvic wrapping | N | 5 |
|  | Immediate cooling care for burns | N | 5 |
|  | Fasciotomy for compartment syndrome | N | 5 |
|  | Intraosseous access | N | 2 |
|  | Needle decompression | N | 2 |
| Dangerous fever | Therapeutics for sympathomimetic toxidromes or ethanol withdrawal | N | 5 |
